# Supplementary material for: Transcriptome sequencing of Verticillium dahliae from a cotton farm reveals positive correlation between virulence and tolerance of sugar-induced hyperosmosis
Source: PeerJ. 2019 Nov 11;7:e8035. doi: 10.7717/peerj.8035 (PMC6855202; doi:10.7717/peerj.8035)
Supplement: Table S1 [file peerj-07-8035-s002.doc]

Table S1. The Nit mutants number and types

| Strains | Nit mutant type | | | VCGs |
| --- | --- | --- | --- | --- |
|  | Nit1 | NitM | Nit3 |  |
| SHZ-2 | 4 | 2 | 0 | VCG2 |
| SHZ-4 | 5 | 2 | 0 | VCG1 |
| SHZ-5 | 2 | 1 | 2 | VCG1 |
| SNZ-6 | 4 | 0 | 0 | VCG1 |
| SHZ-8 | 6 | 2 | 0 | VCG1 |
| SHZ-9 | 3 | 1 | 1 | VCG1 |
| SHZ-11 | 4 | 2 | 0 | VCG1 |
| SHZ-13 | 4 | 0 | 0 | VCG2 |
| SHZ-18 | 4 | 0 | 1 | VCG2 |
| SHZ-21 | 3 | 0 | 0 | VCG2 |
| Total | 39 | 10 | 4 | 53 |
