# Supplementary figures and images for: Transcriptome sequencing of Verticillium dahliae from a cotton farm reveals positive correlation between virulence and tolerance of sugar-induced hyperosmosis
Source: PeerJ. 2019 Nov 11;7:e8035. doi: 10.7717/peerj.8035 (PMC6855202; doi:10.7717/peerj.8035)

# GO enrichment

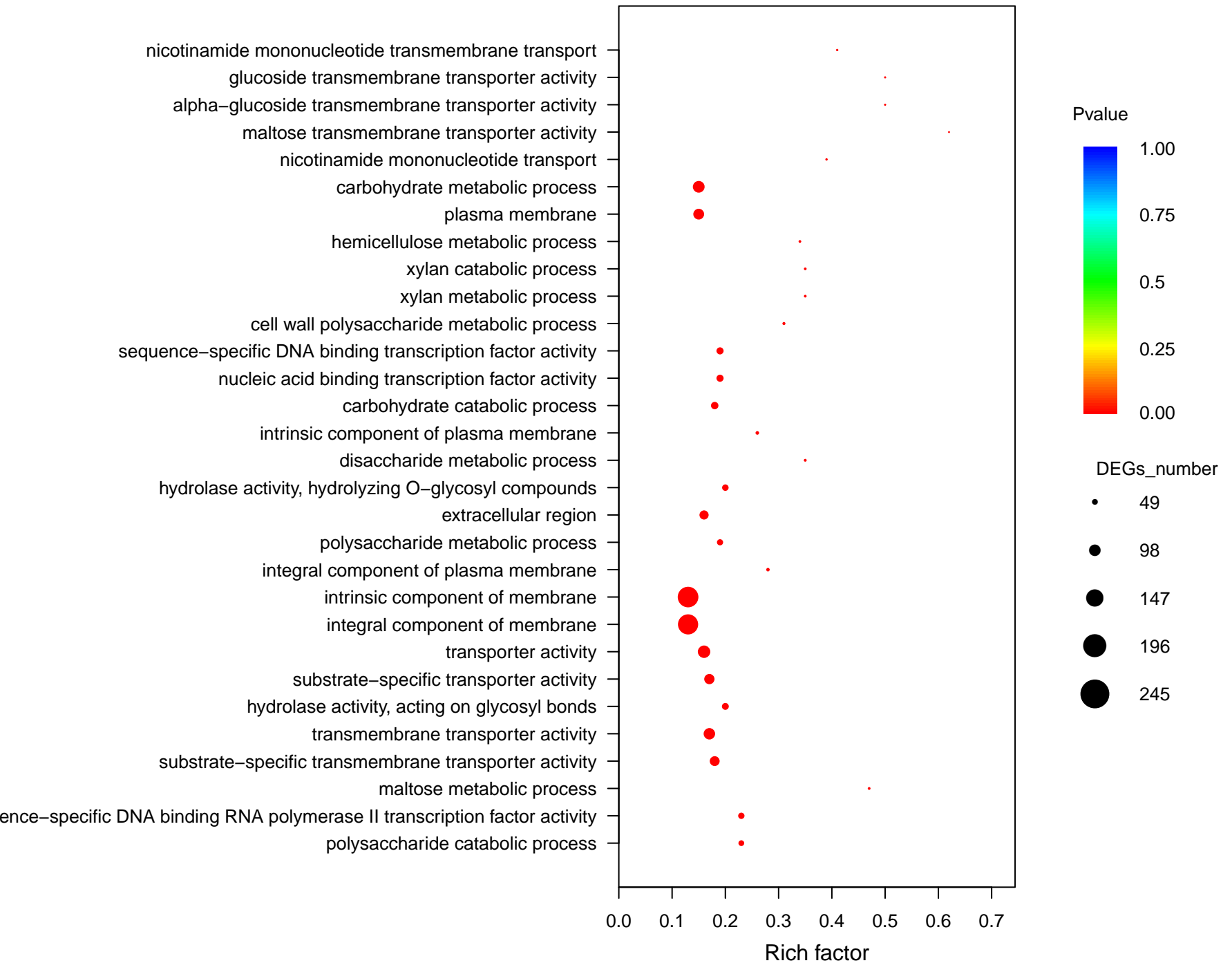

Supplement: Figure S1 [file peerj-07-8035-s006.pdf]

# GO enrichment

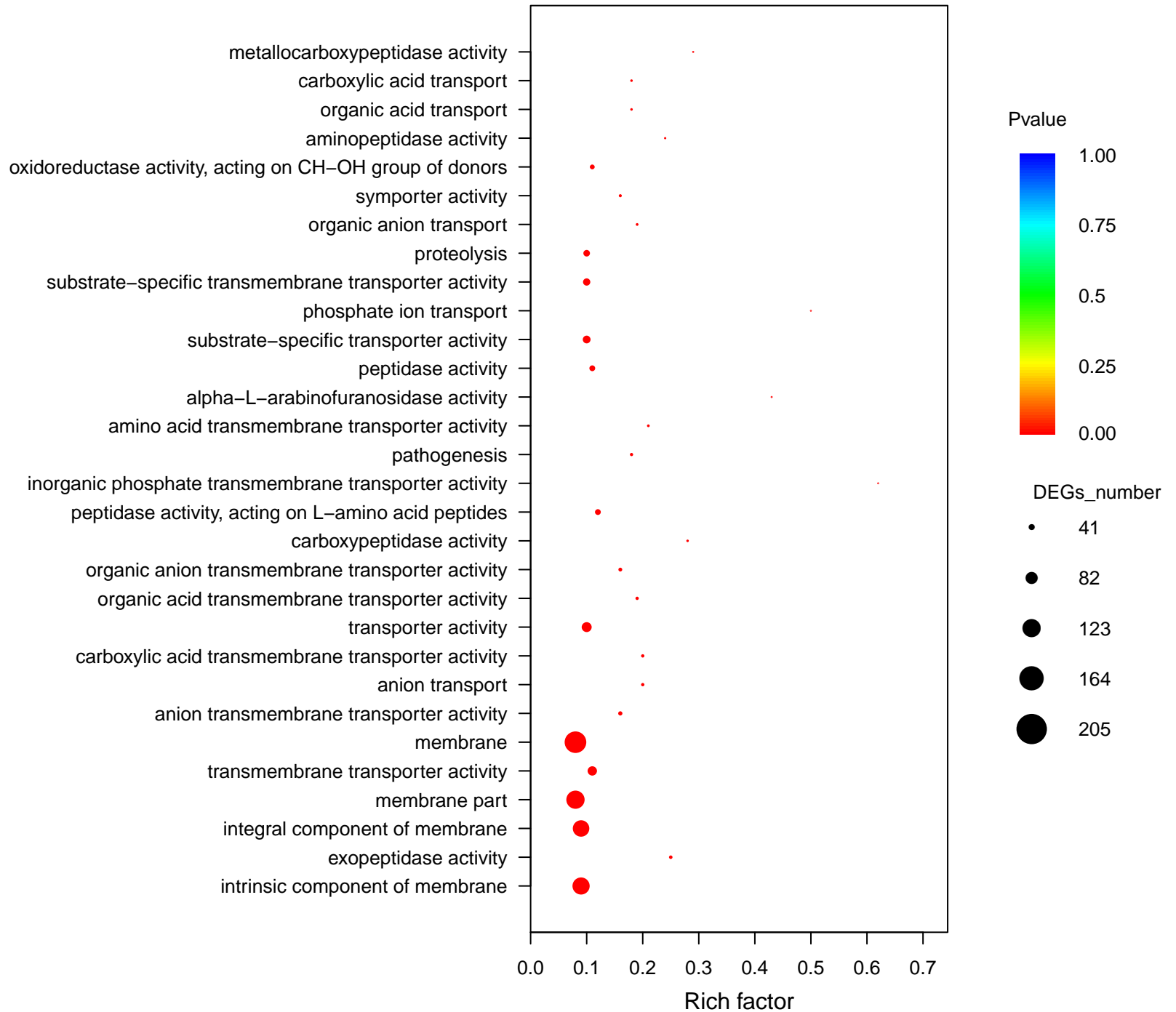

Supplement: Figure S2 [file peerj-07-8035-s007.pdf]

GO enrichment

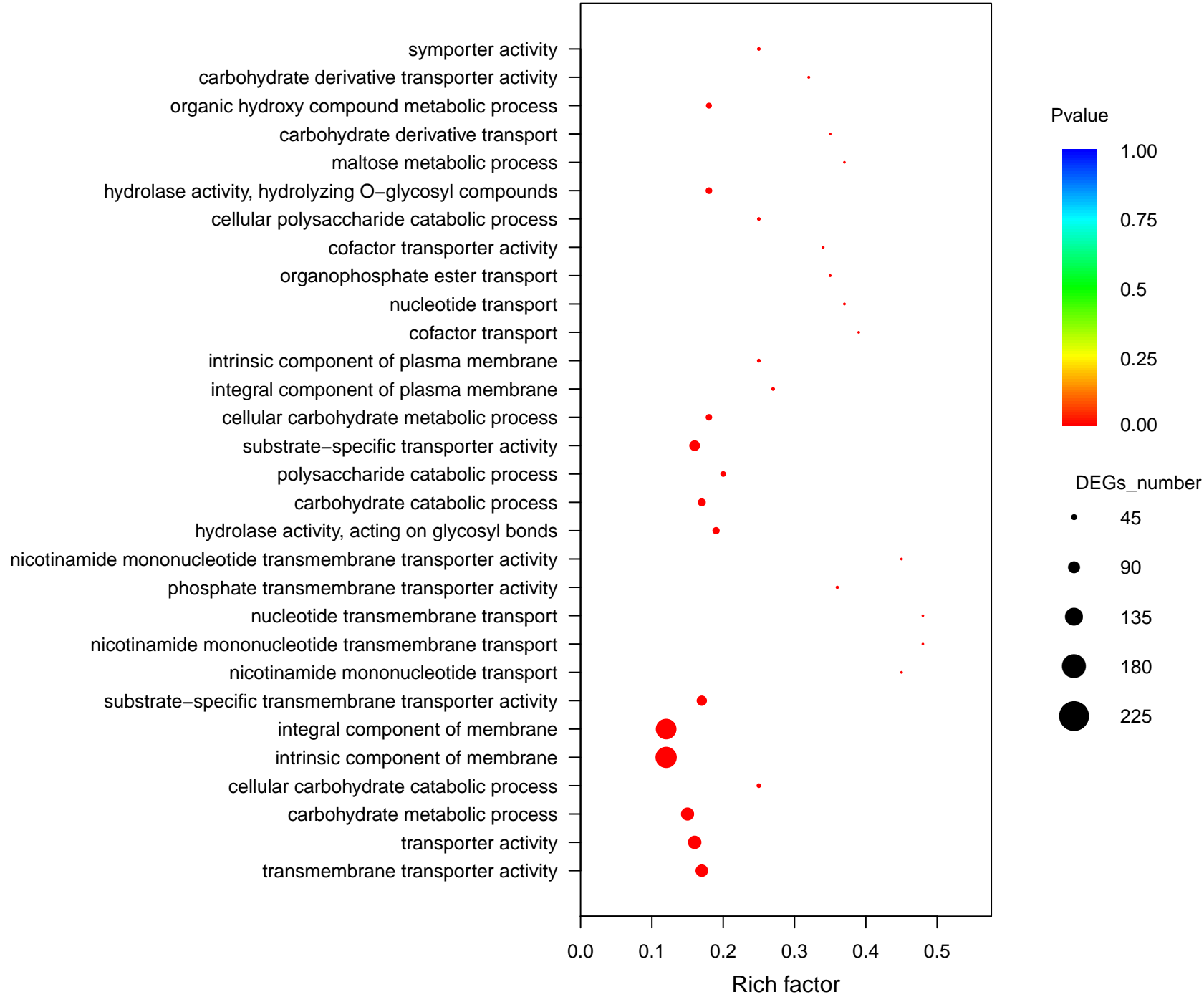

Supplement: Figure S3 [file peerj-07-8035-s008.pdf]
